# Caloric restriction improves health and survival of rhesus monkeys

**DOI:** 10.1038/ncomms14063

**Published:** 2017-01-17

**Authors:** Julie A. Mattison, Ricki J. Colman, T. Mark Beasley, David B. Allison, Joseph W. Kemnitz, George S. Roth, Donald K. Ingram, Richard Weindruch, Rafael de Cabo, Rozalyn M. Anderson

**Affiliations:** 1 Translational Gerontology Branch, National Institute on Aging, Baltimore, Maryland 21224, USA; 2 Wisconsin National Primate Research Center, University of Wisconsin-Madison, Madison, Wisconsin 53715, USA; 3 Department of Biostatistics, University of Alabama, Birmingham, Alabama 35294, USA; 4 Geriatric Research Education and Clinical Center, Birmingham/Atlanta Veterans Administration Hospital, Birmingham, Alabama 35233, USA; 5 Department of Cell and Regenerative Biology, University of Wisconsin-Madison, Madison, Wisconsin 53792, USA; 6 GeroScience, Pylesville, Maryland 21323, USA; 7 Pennington Biomedical Research Center, Baton Rouge, Louisiana 70808, USA; 8 Department of Medicine, University of Wisconsin, Madison, Wisconsin 53792, USA; 9 Geriatric Research Education and Clinical Center, William S. Middleton Memorial Veterans Hospital, Madison, Wisconsin 53705, USA

## Abstract

Caloric restriction (CR) without malnutrition extends lifespan and delays the onset of age-related disorders in most species but its impact in nonhuman primates has been controversial. In the late 1980s two parallel studies were initiated to determine the effect of CR in rhesus monkeys. The University of Wisconsin study reported a significant positive impact of CR on survival, but the National Institute on Aging study detected no significant survival effect. Here we present a direct comparison of longitudinal data from both studies including survival, bodyweight, food intake, fasting glucose levels and age-related morbidity. We describe differences in study design that could contribute to differences in outcomes, and we report species specificity in the impact of CR in terms of optimal onset and diet. Taken together these data confirm that health benefits of CR are conserved in monkeys and suggest that CR mechanisms are likely translatable to human health.

## Abstract (web summary)

Caloric restriction (CR) delays ageing of model organisms, but whether it works in nonhuman primates has been controversial. Here, the authors pool and reanalyse data from two long-running CR primate studies, concluding that moderate CR indeed improves health and survival of rhesus monkeys.

The rhesus monkey (*Macaca mulatta*) is an excellent model for human ageing. The rhesus monkey genome shares ∼93% sequence identity with the human genome^1^,^2^, and numerous aspects of their anatomy, physiology, neurology, endocrinology and immunology directly parallel those of humans^3^,^4^. Rhesus monkeys have a lifespan measured in decades, and develop, mature and age in similar ways to humans. In terms of ageing this includes greying and thinning of hair, redistribution of body fat, loss of skin tone, loss of vigour and loss of muscle tone^4^,^5^,^6^,^7^. With age there are increases in clinical manifestations of diseases and disorders that also increase in prevalence with advancing age in humans, including diabetes, neoplasia, sarcopenia, bone loss, altered immune function and cognitive decline^3^,^4^,^8^. Like humans, nonhuman primates are genetically heterogeneous so that phenotypes of ageing are non-uniformly manifested among individual animals. Variance can be somewhat offset through comprehensive life-long physical and medical health records, where age-related changes are viewed from the perspective of each animal individually. Nonhuman primates exhibit feeding patterns and sleeping behaviour similar to those of humans, and a key advantage of nonhuman primate studies over human studies is that all variables including the environment and dietary intake can be fully defined. In short, nonhuman primates are vital models for translating basic research into clinical application.

A clear understanding of the biology of ageing, as opposed to the biology of individual age-related diseases, could be the critical turning point for novel approaches in preventative strategies to facilitate healthy human ageing. Caloric restriction (CR) offers a powerful paradigm to uncover the cellular and molecular basis for the age-related increase in overall disease vulnerability that is shared by all mammalian species. CR extends median and maximum lifespan in most strains of laboratory rodents and also delays the onset of age-related diseases and disorders^9^,^10^,^11^,^12^. Lifespan is also extended by CR in most short-lived species, including the unicellular yeast, nematodes and invertebrates. There has been rapid progress in identifying potential mechanisms of CR utilizing these models^13^,^14^,^15^,^16^,^17^. These short-lived species are well suited for the investigation of the underlying mechanisms of CR due to the relative ease in their genetic manipulation, extensive genetic and developmental characterization, low cost, and significantly reduced timeframe for completion of longevity studies. A key question underpinning this body of work is whether the biology of CR, and its ability to delay ageing and the onset of disease, applies to humans and human health.

To date three independent studies of rhesus monkeys (*Macaca mulatta*) have tackled the question of translatability of CR to primate species. The University of Maryland rhesus monkey study was the first to report a positive association of CR with survival with a 2.6-fold increased risk of death in control animals compared to restricted^18^. The primary focus of the study was not CR however, and analysis was based on comparing 109 *ad libitum* fed males and females from colony records at that facility, including insulin resistant and diabetic animals, to only eight male CR monkeys. Two other studies focused specifically on the impact of CR in healthy male and female rhesus monkeys: one at the National Institute on Aging (NIA) involving 121 monkeys; and the other at the University of Wisconsin Madison (UW) with 76 monkeys. The same University of Alabama at Birmingham based statistical team was engaged for analysis of data from both studies. The UW study has reported beneficial effects of CR, including significant improvements in health and age-related survival^19^, and all-cause survival^20^. In contrast, the NIA study reported no significant impact of CR on survival, although improvements in health were close to statistical significance^21^. The basis for the contrasting outcomes from these two parallel studies has not been established. Analysis of limited published bodyweight data indicated that the controls were not equivalent between the two studies^20^, pointing to fundamental differences in study design and implementation. Therefore, to more fully assess possible explanations for the discrepant findings between the two studies, we have conducted a comprehensive assessment of longitudinal data from both sites highlighting differences that may have contributed to the dissimilar outcomes.

## Results

### Intrinsic differences in study design

Most of the early rodent CR studies involved very young onset life-long CR initiated post-weaning, usually in inbred genetic backgrounds. In the 1980s it became clear that adult onset CR (12-month-old mice) was also effective in delaying ageing and extending lifespan in rodents, albeit to a lesser extent than the young onset model^22^. Many rodent CR studies opt to feed control animals *ad libitum* amounts of food while others provide less than *ad libitum* amounts arguing that this strategy avoids the confounding effects of obesity and reduces variability in food intake among individuals. With the launch of the NIA rhesus monkey study in 1987, the implementation of CR was such that the control monkeys were not free-fed. Food allotments were determined in accordance with data published by the National Research Council to provide approximate *ad libitum* intake based on their age and bodyweight for the maturing control monkeys without overfeeding^23^. Rations were increased to maintain growth and development until full stature was attained. CR monkeys received 30% less food than height-, age- and sex-matched control monkeys. The intervention was initiated as young-onset and old-onset groups of males, and young, adult, and old-onset groups of females^24^ (Table 1). Launched in 1989, the UW study initiated the CR diet in adult animals only, after full stature was achieved (∼8 years of age for rhesus monkeys)^25^. Food was provided at levels approximating *ad libitum* to control animals. To accommodate heterogeneity in the feeding behaviours within the cohort, the *ad libitum* reference for each individual was established using baseline food intake measured over 3–6 months, and CR was implemented on a per-individual basis. The rationale for these design features at UW was to implement a study as it might have been conducted in humans.

The source of the monkeys in each cohort and the population type represented is also a point of difference for the two studies. The UW monkeys were born and raised at the Wisconsin National Primate Research Center and were all of Indian origin. The NIA monkeys were sourced from several locations and included monkeys of both Indian and Chinese origin. Chinese male rhesus monkeys are generally heavier and longer than their Indian counter parts with the reverse being the case for females, and Chinese rhesus monkeys are also thought to exhibit greater sexual dimorphism^26^. Monkeys of different origin are sufficiently genetically different that they can be distinguished using a panel of single nucleotide polymorphisms^27^. Apart from population differences, rhesus monkeys share a similar degree of inter-individual genetic variation as humans^28^. In this way, the contribution of population type to differences in outcomes of the two studies as opposed to the contribution of individual genetic heterogeneity is difficult to ascertain.

The diet compositions were another important difference between the two studies. First, the source of diet components was different. A naturally sourced diet was employed at the NIA facility to ensure that micronutrients such as phytochemicals and trace minerals were provided, acknowledging that there was potential for seasonal variation. In contrast, a semi-purified diet was employed at UW to ensure that intake could be fully defined and consistent throughout the course of the study. Second, although diets at both locations had a similar caloric density, the relative macronutrient composition of the diets was not equivalent (Table 2). Compared to the UW diet, the NIA diet was lower in fat, higher in protein and higher in fibre. Finally, the nutrient content of the diets was also different. At both locations diets contained∼60% carbohydrates by weight, but sucrose comprised less than 7% of total carbohydrates at NIA and 45% of total carbohydrates at UW. Diets at both locations were replete for vitamins that were provided at or above the recommended daily allowance.

Feeding practices also differed between studies. At NIA, the monkeys were fed two meals at ∼6:30 and 13:00 each day. Any food remaining after the morning meal was removed after about 3 h, and a low calorie treat was provided, typically in the form of a small piece of fruit. The afternoon meal was not removed so that monkeys had access to food at night. At UW, all monkeys were fed in the morning at∼8:00 and any remaining food was removed at ∼16:00 when a treat of fresh fruit or vegetable, which was quickly and completely eaten, was provided. Food allotment for control animals was adjusted to ensure that there was always some uneaten food to be removed at the end of the day. In this way UW animals were *ad libitum* fed during the day but food deprived overnight. While there were considerable differences in study design as outlined above, it should be noted that animal housing and routine animal care were equivalent at NIA and UW primate facilities. This included identical housing conditions, temperature and humidity range, light cycles, and the use of tap water, which was continuously available. Both studies included animal monitoring several times per day, and a designated veterinary staff that inspected the animals routinely and provided outstanding care as needed.

### Impact of CR on survival

**Figure 1:**
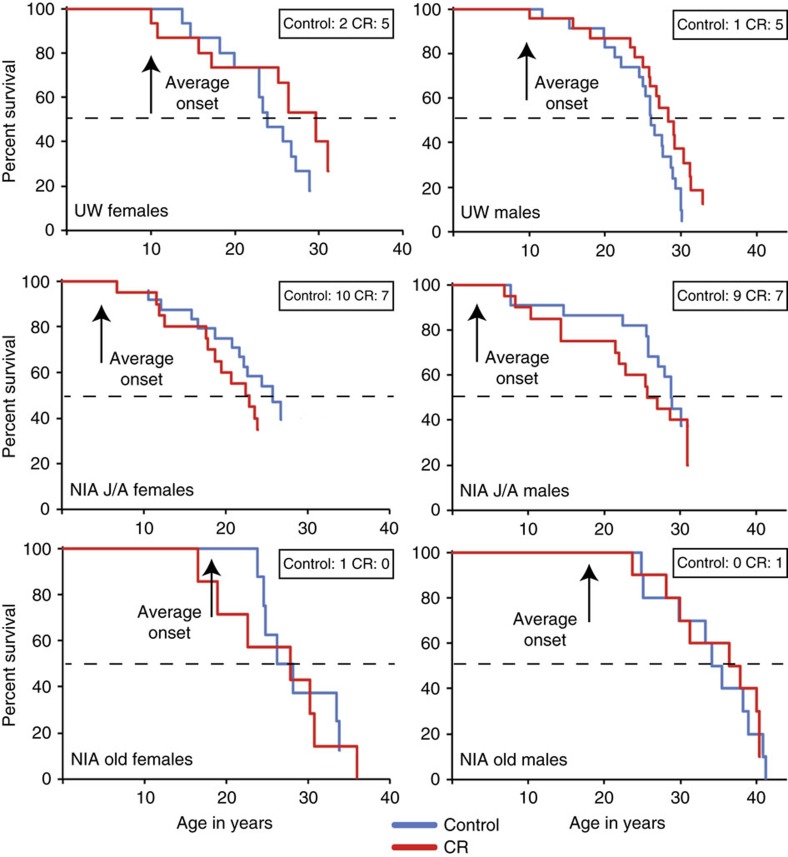
Mortality curves for monkeys at UW and at NIA. These curves depict data for male and female monkeys on the UW study and on the NIA study. Animals are grouped by age where male J/A include juvenile and adolescent onset animals, female J/A include juvenile and adult onset animals, and old include the advanced age onset animals. Inset boxes indicate animals still alive, dashed line marks 50% mortality. Statistics related to this figure are provided in Supplementary Information, Supplementary Table 1.

The initial goal of both NIA and UW studies was to determine the impact of CR on the health of rhesus monkeys, as it was not a foregone conclusion that CR would be an appropriate intervention in long-lived species. The investigation of the impact of CR on longevity was not considered a primary outcome at either study location. Even though 121 monkeys were enrolled in the NIA study, the differences in age of onset (from 1 to 23 years) precluded the animals from being grouped together for data analyses. Although the age range for time of onset is smaller for the UW study (ages 7–15), with only 38 outbred genetically distinct monkeys per group (including both sexes), it seemed unlikely that the study would have the statistical power required to test CR's effect on longevity. While neither study reports longevity data, both studies have yielded survival data. For rhesus monkeys in captivity, the previously reported median survival was ∼26 years of age, 10% survival was ∼35 years of age and maximal survival was ∼40 years of age^29^. Mortality curves were generated separately for UW and NIA (Fig. 1). Survival estimates for monkeys at both sites were calculated based on data captured up to July 2015 using the three most common statistical methods: Kaplan-Meier product-limit method; Cox proportional hazard regression and parametric survival analysis assuming a Weibull distribution (Table 3). Because the Weibull distribution is a special case of the generalized extreme value distribution, it can accommodate estimation of the upper quantiles of a survival distribution and maximal lifespan, especially when there are censored data due to animals that remain alive^30^.

In the UW adult-onset study, the estimated survival of UW control animals was close to that of the average recorded for monkeys in captivity (∼26 years of age). Considering both males and females together, a statistically significant effect of CR in increasing survival was observed (Cox regression *P*=0.017; Supplementary Table 1). The hazard ratio (HR) of 1.865 (95% confidence interval (CI): 1.119–3.108) indicated that at any time-point the control monkeys had almost twice the rate of death when compared to CR animals. The effect of sex on the response to CR was not statistically significant. Kaplan-Meier analysis showed that median survival estimates were greater for CR animals for both males and females (Table 3). In the NIA study large differences in ages of monkeys at time of recruitment to the study (Table 1) prompted a separation of data from the early and late onset groups. Here and throughout this report, NIA male juveniles and adolescents (J/A) were grouped and female juveniles and adults were grouped (J/A). The Kaplan-Meier median estimated survival was not different between NIA control and CR animals for the J/A onset groups of males or females (Fig. 1). Although Cox proportional hazard regression indicated that the differences in survival between J/A control and CR were not statistically significant (Supplementary Table 1), CR monkeys reached 80% mortality before the controls for both sexes. With 38% of the NIA J/A cohort still alive, the survival curves are incomplete and the impact on survival remains to be determined; however, the early mortality suggests that for some individuals implementation of CR in the very young may confer a survival risk. For old-onset CR, Kaplan-Meier estimated survival was not different between control and CR groups for either males or females (Table 3), but survival estimates were higher than those of J/A monkeys and UW controls. For both males and females, survival estimates for the NIA old-onset cohort were comparable to or exceeded those for UW CR.

Although there were slight discrepancies in the estimated median survival between the non-parametric Kaplan-Meier and parametric Weibull estimation methods, the survival comparisons between study sites using either analysis were consistent. A certain degree of sexual dimorphism was observed in survival outcomes where incidence of early death appeared to be greater for females. This observation might be explained in part by endometriosis, which is the proliferation of endometrial tissue outside of the uterus. Endometriosis can occur at relative high incidence in monkeys in captivity (∼25%), and risk is considerably greater for nulliparous females^31^,^32^. Incidence of endometriosis was equivalent for control and CR groups. For the J/A cohorts in the NIA study, 12 of the 44 females died of complications due to endometriosis, and of these the juvenile onset females were confirmed nulliparous. Females recruited to the UW study, in contrast, had at least one but no more than three healthy infants^33^, and only 2 of 30 females died of complications due to endometriosis. A further contributing factor relates to the policy on treatment of clinical conditions. At UW the policy to treat clinical conditions was implemented from the outset. At NIA, although acute pain and suffering were always treated, chronic medical conditions, including endometriosis, were monitored but not medically treated. A policy change was implemented in 2010 due to the high incidence of endometriosis. The power to assess the impact of CR on survival for NIA J/A females has been compromised somewhat by this one condition.

### Biometric and food intake measures from both studies

**Figure 2:**
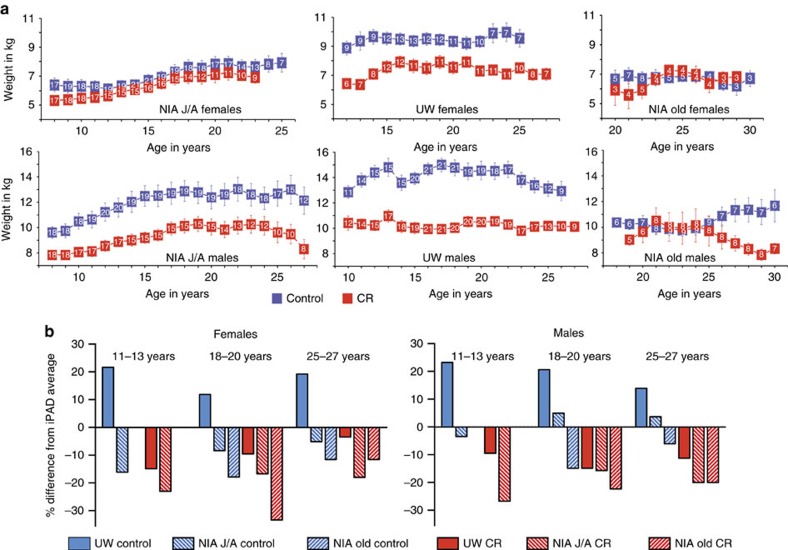
Bodyweight data for monkeys at NIA and UW. (**a**) Bodyweight (kg) for male and female monkeys at UW and at NIA grouped by age where male J/A include juvenile and adolescent onset animals, female J/A include juvenile and adult onset animals, and old include the advanced age onset animals. Digits shown in white within the boxes are the numbers of individual animals contributing to each data point, data are shown as mean±s.e. of the mean. (**b**) Comparison of bodyweight averages for monkeys from UW and NIA studies with records of the internet Primate Aging Database (iPAD). Average bodyweight for control and CR monkeys at both study locations were determined by age category including adult (11–13 years of age), late mid-age (18–20 years of age) and advanced age (25–27 years of age). Data are expressed as percent deviation from the iPAD average for females and males from each age category. Statistics related to this figure are provided in Supplementary Information, Supplementary Tables 2 and 3.

For over a quarter of a century during these studies, bodyweight, body composition and food intake were measured for all 197 monkeys. Bodyweight was determined in fasted and anesthetized monkeys 2–4 times per year during routine procedures. Longitudinal data for all monkeys were averaged by age of the animal (Fig. 2a). As is the case for humans, monkeys often experience cachexia or end-of-life rapid weight loss. To avoid confounding effects of weight change that is not related to food intake or diet, data from the last year of life for each monkey were excluded. To facilitate comparisons among the cohorts, data were grouped into three age categories representing young adult (11–13 years of age), late mid-age (18–20 years of age) and advanced age (25–27 years of age) (Supplementary Tables 2 and 3).

Considering first the female monkeys, bodyweight for the NIA J/A was not significantly different between control and CR monkeys for any age categories. UW CR females weighed significantly less (17–26%) than controls throughout the study period, and UW female controls weighed significantly more than NIA J/A female controls throughout (Supplementary Table 2). For NIA old-onset females, bodyweight was not significantly different between controls and CR, and was significantly lower than bodyweight of UW female controls. In summary, for NIA J/A and old-onset female cohorts, bodyweight for control and CR monkeys was not different from each other and all were significantly lower than the UW controls. Considering next the male monkeys, NIA J/A CR males weighed significantly less (19–22%) than their control counterparts throughout the study. The difference between UW control and CR was slightly greater (24–35%), with CR males weighing significantly less than controls. The average peak weight for NIA J/A control males was ∼15% lower than that of UW control males, but differences in bodyweight were significant for the young age category only (Supplementary Table 3). Bodyweight of the old-onset NIA control and CR males were not significantly different at either mid-age or advanced ages, and old-onset NIA male controls weighed significantly less than UW controls. In summary, NIA J/A and UW male cohorts showed a clear bodyweight response to CR, but old-onset NIA control and CR males were not different from each other and were significantly lower than the UW controls.

The internet Primate Aging Database (iPAD; http://ipad.primate.wisc.edu) is a repository of clinical and biometric data from healthy, non-experimental, captive nonhuman primates housed at research facilities across the USA. Using data from over 1,200 individual rhesus monkeys of Indian origin, mean bodyweights were calculated for the above age categories for males (11.6, 12.1, 11.5 kg respectively) and females (7.4, 8.4, 7.8 kg respectively). UW control and CR monkeys fell on either side of these averages; control monkeys were heavier than the iPAD average (∼18% for males; ∼19% for females), and CR monkeys had lower bodyweight than the iPAD average (∼12% for males; ∼11% for females) (Fig. 2b). For NIA J/A, control males were the same to slightly heavier (5–10%) than the iPAD average and CR weighed less than the iPAD average (∼20%), while control and CR female monkeys both weighed less than the iPAD average throughout the study (∼10% and ∼20% respectively). All NIA old-onset monkeys weighed less than the iPAD average for both control (∼15% for females; ∼10% for males) and CR (∼22% for females; ∼21% for males) monkeys. In summary, bodyweights of UW and NIA control monkeys were not equivalent to each other, and apart from J/A males, were respectively higher and lower of the iPAD average.

**Figure 3:**
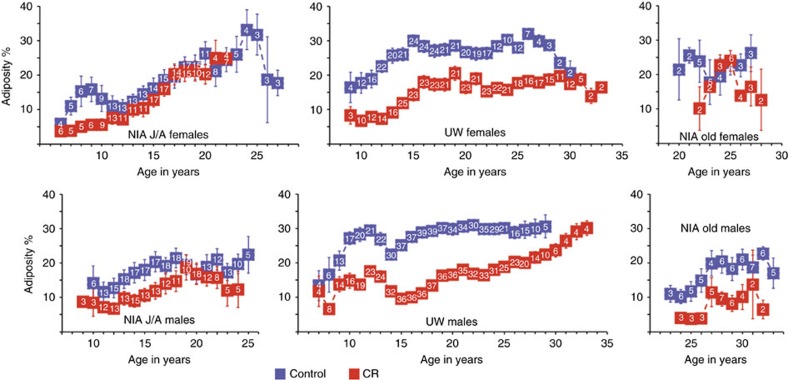
Adiposity data for female and male monkeys at NIA and UW. Percent adiposity (fat (g)/total bodyweight (g)) calculated from DXA (dual energy X-ray absorptiometry) measures conducted during the course of the studies for male and female monkeys at UW and at NIA grouped by age where male J/A include juvenile and adolescent onset animals, female J/A include juvenile and adult onset animals, and old include the advanced age onset animals. Digits shown in white within the boxes are the numbers of individual animals contributing to each data point, data are shown as mean±s.e. of the mean.

To gain insight into differences in the effect of age and diet on body composition, dual X-ray absorptiometry measures were conducted at intervals throughout the course of the two studies (Fig. 3). Since each animal had multiple measures taken over time, estimates of the average percent adiposity (fat/bodyweight expressed as percent) were adjusted for age (Supplementary Fig. 1). Within groups a main effect of age on adiposity was detected for NIA J/A and UW cohorts. A main effect of diet was detected for NIA J/A males and for both males and females from the UW study, where CR was associated with significantly lower adiposity. The NIA J/A control and CR females did not differ from each other in adiposity and neither of the NIA old-onset monkey groups had a main effect of CR on adiposity. Combining the data from NIA J/A and UW, a difference in adiposity was detected between controls on the two studies for both males and females, where NIA monkeys had significantly lower percent body fat. Control monkeys from NIA J/A were not statistically different from UW CR in percent body fat for both sexes. These data show an impact of age on adiposity in all three groups and reveal that the impact of CR on adiposity was observed for both groups of UW monkeys and at NIA for J/A males only.

**Figure 4:**
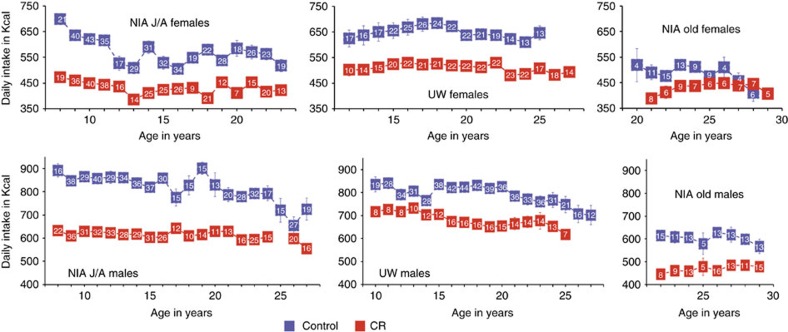
Food intake data for monkeys at NIA and UW. Food intake (daily values in Kcalories) for male and female monkeys at UW and at NIA grouped by age where male J/A include juvenile and adolescent onset animals, female J/A include juvenile and adult onset animals, and old include the advanced age onset animals. Digits shown in white within the boxes are the numbers of individual animals contributing to each data point, data are shown as mean±s.e. of the mean.

Food intake was monitored daily at both sites. At UW daily measures of food intake were used to calculate means. At NIA food intake means were calculated based on measures conducted during a single week per year as representative of typical intake. Longitudinal data for all monkeys were averaged by age of the animal (Fig. 4). Data from the last year of life of each monkey were excluded to avoid confounding effects of end-of-life feeding behaviours that usually include loss of appetite. Considering first the females and using the age categories defined above for both UW and NIA J/A, the controls consumed significantly more calories than CR at both young and mid-age, but the difference persisted only for UW female monkeys at advanced age. For the old-onset NIA, caloric intake was not different between control and CR. Among control monkeys, UW females consumed significantly more calories than NIA J/A at mid-age and advanced age and more than old-onset at advanced age. Considering next the males, the NIA J/A controls consumed significantly more calories than CR at young and mid-age and the difference between control and CR was significant for UW at mid-age only. Old-onset males at NIA differed significantly in their caloric intake between control and CR only at advanced age. Among controls, caloric intake was not different for NIA J/A and UW males at any point in the study, but old-onset males consumed significantly less than UW males and NIA J/A males at mid-age. In summary, significant differences in caloric intake were identified between control and CR monkeys for male and female NIA J/A and UW cohorts, but not for old-onset cohorts until advanced age and then for males only. Comparing between sites, caloric intake for NIA female controls of both J/A and old-onset was lower than that of UW controls, and for males, caloric intake of NIA J/A and UW controls were not different from each other but old-onset NIA controls were lower than both.

### Impact of CR on incidence of disease

The concept of healthspan is a fairly recent development in ageing research, where a distinction is drawn between chronological age and health status^34^. Traditionally, an increase in both median and maximum lifespan was considered the hallmark of delayed ageing, and improvements in health were deemed to be a necessary and obvious component of longevity. The perspective has shifted somewhat towards greater emphasis on health and morbidity, so an intervention that imparts improved health even in the absence of increased longevity, is viewed as a highly favourable and legitimate example of an ageing intervention. With advancing age, rhesus monkeys are vulnerable to many of the same conditions observed in humans. Among the most prevalent are cancer, cardiac disease, and conditions related to immune dysfunction and inflammation, and examples of each were identified in monkeys on the ageing and CR studies at both NIA and UW (Supplementary Table 4).

**Figure 5:**
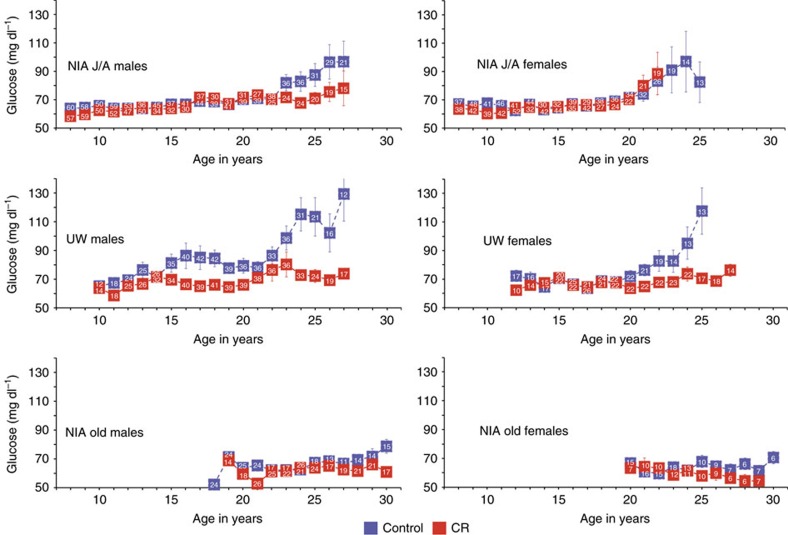
Fasting glucose values for monkeys at NIA and UW. Circulating levels of glucose (mg dl^−1^) are shown for male and female monkeys at UW and at NIA grouped by age where male J/A include juvenile and adolescent onset animals, female J/A include juvenile and adult onset animals, and old include the advanced age onset animals. Digits shown in white within the boxes are the numbers of observations contributing to each data point, data are shown as mean±s.e. of the mean.

Fasting glucose measures were common to both studies and the longitudinal data are shown (Fig. 5). In healthy adult rhesus monkeys fasting glucose levels are 64–68 mg dl^−1^ (refs 18, 35). For NIA J/A, fasting glucose levels were equivalent for controls and CR up to ∼23 years of age, after which the control and CR males, but not females, began to diverge. Both control and CR females showed an age-related increase in fasting glucose levels after ∼21 years of age. For UW monkeys, the control males had higher fasting glucose levels than CR from 15 years of age with a further divergence of the curves after ∼23 years of age, while a noticeable difference between control and CR females emerged after only ∼21 years. For the NIA old-onset cohorts, fasting glucose was consistently low for the duration of the study period. These data point to an age-related increase in fasting glucose for rhesus monkeys and single out the UW control males as being predisposed to elevated circulating glucose in the fasted state. Using multilevel modelling to investigate the relationship between adiposity and fasting glucose levels a significant relationship was identified for UW males only (*P*=0.005). A significant age by diet interaction was also detected (*P*=0.014), suggesting that the impact of age on the relationship between adiposity and glucoregulatory parameters is distinct for control and CR monkeys.

**Figure 6:**
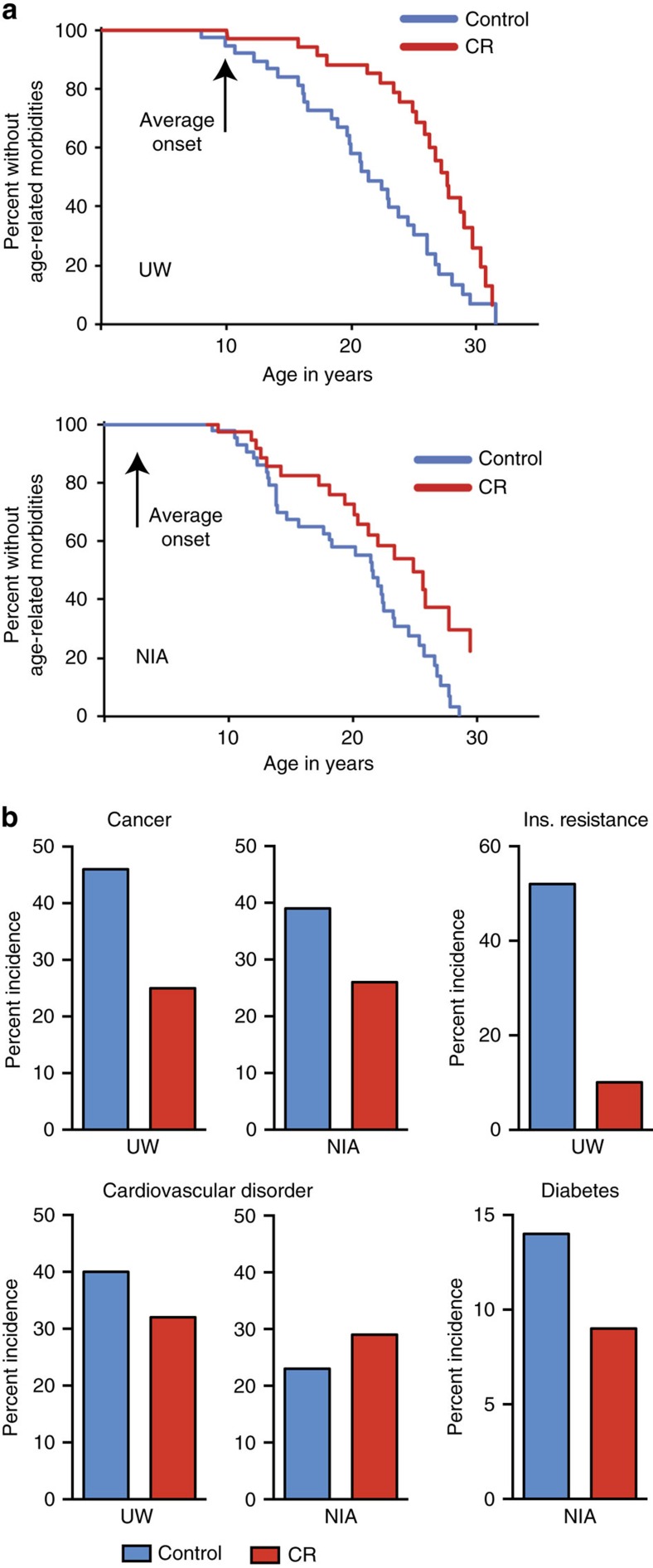
Morbidity curves for monkeys at NIA and UW shown. (**a**) Graphs represent the first occurrence of any age-related disease, disorder or condition for combined males and females from UW (top) and NIA J/A (bottom). Statistics related to this figure are provided in Supplementary Information, Supplementary Table 4. (**b**) Incidence of prevalent age-related conditions in nonhuman primates for control and CR animals from UW and NIA (J/A and old-onset combined). To compare studies, cancer and cardiovascular disorders are reported as incidence upon necropsy and are expressed as a percentage of the animals that are deceased.

Veterinarians documented body condition and overall health of monkeys biannually at both study locations and indicators of diseases or disorders identified. The age at which a monkey was first diagnosed with an age-related condition was used to generate morbidity curves (Fig. 6). Age-related conditions included sarcopenia, osteoporosis, arthritis, diverticulosis, cataracts and persistent heart murmurs, in addition to age-related diseases including cancer, diabetes and cardiovascular disease. Cox proportional hazard regression modelling indicated that age-related conditions occurred at ∼2.7 times the rate in control animals compared to CR for UW monkeys (HR: 2.665; CI: 1.527–4.653; *P*=0.0006). In the NIA J/A cohort, age-related conditions occurred at twice the rate in control monkeys compared to CR (HR: 2.091; CI: 1.169–3.641; *P*=0.0125) (Supplementary Table 5; Supplementary Fig. 2). The advanced age of the old-onset NIA monkeys precluded detection of the first occurrence of an age-related condition.

The incidence of age-related conditions prevalent in human populations, such as cancer, cardiovascular disease and glucoregulatory dysfunction/diabetes, was determined for monkeys at UW and NIA, with J/A and old-onset combined. Diagnoses were made clinically by veterinary staff upon presentation, and disease-related pathology was subsequently confirmed upon necropsy by a board-certified pathologist. Clinically silent pathologies were identified at necropsy. To compare studies, cancer and cardiovascular disorders are reported as incidence upon necropsy. Adenocarcinoma was the leading cause of death at both study sites, consistent with previous reports on cancer incidence in rhesus monkeys^36^,^37^. The incidence of adenocarcinoma or other less common neoplasms was lower in CR monkeys at both locations (Fig. 6). The most common diagnosis of cardiovascular disease in live animals was mitral valve dysfunction, while valvular endocardiosis, cardiomyopathy and myocardial fibrosis were detected at necropsy. Incidence of cardiovascular disorders was lower in CR monkeys than controls at UW. There was no apparent impact of diet on incidence of cardiovascular disease for monkeys at NIA; however, incidence for both control and CR monkeys was lower than UW controls. Assessment of glucoregulatory function was part of routine clinical care at both locations, although clinical definitions representing different disease stages were employed at the two sites. Similar to humans^38^,^39^, insulin resistance occurs in advance of impaired fasting glucose in rhesus monkeys^35^,^40^, which occurs before transition to full diabetes. At UW loss of insulin sensitivity was used to diagnose glucoregulatory impairment and was defined as fasting insulin levels (>70 μU ml^−1^) and an insulin sensitivity index (S_i_)<2(E^−04^) as determined by a frequently sampled intravenous glucose tolerance test. At NIA, fasting glucose (>100 mg dl^−1^), glucosuria and HbA1c (>6.5%) measures were used to define diabetes. CR animals had lower incidence of glucoregulatory dysfunction than controls at both UW and NIA study sites. Multilevel modelling was used to investigate possible relationships between adiposity and morbidity but no significant effects were detected for any group from either study. Similarly, for all NIA groups and for UW males, no relationship between adiposity and mortality was detected. For UW females, adiposity was associated with a modest reduction in risk for death (HR: 0.927; *P*=0.01) but only after correcting for age and diet. These data suggest that the influence of adiposity on survival risk is sexually dimorphic and changes with age.

## Discussion

Data from both study locations suggest that the CR paradigm is effective in delaying the effects of ageing in nonhuman primates but that the age of onset is an important factor in determining the extent to which beneficial effects of CR might be induced. In the UW study, reduced bodyweight, reduced adiposity and reduced food intake of the CR monkeys were associated with improved survival, with CR monkeys of both sexes surviving longer than controls, ∼28 and ∼30 years of age for males and females respectively, and longer than the median age for monkeys in captivity (∼26 years of age). Although an impact of CR on survival was not detected within the NIA old-onset cohort, comparison to the UW study shows that bodyweight was significantly lower in both control and CR groups of males and females than in their UW control counterparts, and was largely equivalent to that of UW CR. All males and females from the NIA old-onset groups consumed fewer calories than their counterpart controls from UW, instead both control and CR were closely aligned with food intake values of UW CR. Importantly, the median survival estimates for old-onset males were very high, similar to what has been reported previously as the 90th percentile for this species (∼35 years of age). Six of the original 20 monkeys have lived beyond 40 years of age, the previous maximal lifespan recorded, and one old-onset CR male monkey is currently 43 years old, which is a longevity record for this species. Median survival estimates for old-onset females, ∼27 and ∼28 for controls and CR respectively, were also greater than national median lifespan estimates, with one remaining female currently 38 years of age. The clear benefit in survival estimates for monkeys within the old-onset cohort compared to UW controls suggests that food intake can and does influence survival. The lack of difference between control and CR old-onset monkeys suggests that a reduction in food intake beyond that of the controls brings no further advantage. The minimum degree of restriction that confers maximal benefit in rhesus monkeys has not yet been identified but is an active topic of investigation. Taken together, data from both UW and NIA studies support the concept that lower food intake in adult or advanced age is associated with improved survival in nonhuman primates.

The interpretation of the outcomes from NIA J/A cohorts is more complicated. The J/A cohorts reveal a sex-dependence in the relationship between bodyweight and food intake that was not observed in the UW cohorts. The NIA J/A CR males consumed significantly fewer calories than controls for most of the study, and this was reflected in a significantly lower bodyweight and a modest reduction in adiposity. Bodyweight of the NIA J/A control males was in between that of UW controls and CR monkeys even though caloric intake was equivalent between UW and NIA control animals, and age-adjusted average adiposity of NIA J/A males was equivalent to that of UW CR males. Median survival estimates for the NIA J/A male controls and CR were not statistically different at ∼29 and ∼26 years, respectively, but estimates for the J/A controls were numerically equivalent to UW CR. These data suggest that in the J/A male cohort, the NIA diet is associated with lower bodyweight, lower adiposity and improved survival in the absence of CR, and that no further advantage is gained by lowering food intake or bodyweight below that of controls. A different picture emerges from the data from NIA J/A females. The CR monkeys consumed significantly less food than controls for much of the study, but there was no apparent difference in bodyweight or adiposity between them. Median survival estimates for control and CR J/A females were not statistically different from each other at ∼26 and ∼23 years respectively, and each were lower than that observed for UW CR. The lack of response in bodyweight to differences in food intake in the female monkeys suggests that there is sexual dimorphism in the relationship between food intake and bodyweight in young animals and in how these parameters relate to median survival. Finally, there is a suggestion that nonhuman primates differ from rodents in the suitability of early onset CR. In rodents, early onset CR is more effective in extending longevity than adult onset CR. For nonhuman primates it appears that CR, while beneficial when implemented in adulthood, does not improve survival when implemented in juveniles. Furthermore, it appears that CR is not uniformly tolerated in young animals, where the NIA J/A CR males and females reached 80% mortality earlier than controls. Although the numerical values for estimated survival were lower in CR groups than in controls, the impact of juvenile onset CR on survival remains undetermined, as the mortality curves for both NIA J/A cohorts are incomplete.

Data from both NIA and UW studies highlight sex-dependent differences in nutritional modulation of body composition and glucoregulatory function, and prompt further investigation of sex-specificity in the connection between nutrition and disease risk. In rodent studies sexual dimorphism in the response to diet-induced metabolic dysfunction has been reported where males were more vulnerable than females to increased adiposity and glucoregulatory dysfunction^41^. It is perhaps not surprising then that the separation in fasting glucose curves between UW controls and CR was observed in males but not females, and that females, despite being on the same diet as males, were not different in glucose levels between control and CR until after 20 years of age. Although both NIA and UW diets contain equivalent relative amounts of carbohydrate, the sucrose content of the purified UW diet is six times higher than the naturally sourced NIA diet, and the fat content of the UW diet is twice that of the NIA diet. The implication is that elevated sucrose and increased fat content could lead to increased adiposity, negatively impacting glucoregulatory function. All the NIA J/A monkeys maintained adiposity at less than 25% and were in large part euglycemic through middle age. As might be predicted, the UW control males and females had significantly greater adiposity compared to NIA controls. In the case of UW males, increased adiposity was significantly associated with higher fasting glucose levels; however, the association did not hold for the UW female monkeys where despite differences in adiposity between control and CR, fasting glucose levels were equivalent through middle age, and multilevel analysis failed to identify a significant association between adiposity and fasting glucose. Furthermore, there is the suggestion that the effect of diet composition is dependent on intake levels. The UW CR male monkeys, on the same diet as controls but under food limiting conditions, had low adiposity and normal fasting glucose. These data demonstrate that the relationship between adiposity and glucoregulatory impairment is sex-specific, and the relationship between dietary composition and glucoregulatory impairment is dependent on the level of food intake. The significant relationship between adiposity and survival detected in the UW female cohort also points to sex-dimorphism in the impact of age and the influence of body composition on mortality risk. This relationship was identified for UW females only, presumably because there was a greater range in levels of adiposity among controls and CR for the UW females than for NIA females where adiposity of monkeys from either diet at either age of onset did not differ. The positive relationship between adiposity and survival in UW females was identified only after correcting for age and diet, suggesting that the interactions among health, body composition and morbidity are sensitive to age and, given the lack of association in the male cohort, sex-dependent.

The catalogue of pathologies identified in aged monkeys is shared with aged humans. The definitions used to identify morbidity were determined by veterinary staff and were essentially equivalent at both sites. A shared feature of both studies is the beneficial effect of CR in lowering the risk for age-related morbidity by more than two-fold. Factors contributing to these analyses include a range of conditions that are highly prevalent in human geriatric populations, such as sarcopenia, osteoporosis and arthritis. The beneficial effects extended to diseases that are among the most prevalent in human clinical care including cancer, cardiovascular disease and parameters associated with diabetes. A lower incidence of cancer was one of the first health benefits of CR documented and is considered to be a hallmark of CR in rodents^11^. The incidence of cancer was lower in CR monkeys at both locations indicating that tumour suppression is a conserved feature of mammalian CR. CR also lowered the incidence of cardiovascular disorders at UW, and NIA monkeys from either diet group appear to have been protected compared to UW Control monkeys. The data shown are based on pathologies identified at necropsy so that final incidence of both disorders at NIA has yet to be determined. The impact of CR to sustain glucoregulatory function during ageing was a further commonality between the studies. Monkeys presenting with insulin resistance at UW were clinically treated to prevent transition to diabetes, prohibiting a direct comparison between the studies. Although the diagnosis employed at the two study locations represent different time frames in disease progression, the outcome of lower incidence of insulin resistance at UW and lower incidence of diabetes at NIA are very much in agreement. Given the obvious parallels between human and rhesus monkey, it seems highly likely that the beneficial effects of CR would also be observed in humans. Reports from the multicenter CALERIE study of short-term CR in humans document changes in bodyweight, body composition, glucoregulatory function and serum risk factors for cardiovascular disease in response to CR (refs 42, 43, 44, 45, 46, 47). These outcomes in humans align well with reports on rhesus monkey CR (refs 48, 49, 50, 51), confirming that the primary response to CR is conserved between these two species, and suggesting that the underlying mechanisms may also be conserved.

In conclusion, the NIA and UW nonhuman primate ageing and CR studies address a central concept of relevance to human ageing and human health: that the age-related increase in disease vulnerability in primates is malleable and that ageing itself presents a reasonable target for intervention. The last two decades have seen considerable advances in ageing research in short-lived species and investigations of the mechanisms of CR have been prominent in this work. It will be particularly informative to determine the degree to which consensus hallmarks of ageing described in recent publications^52^,^53^ also manifest in primate ageing. The tissues and longitudinal data stored over the course of these two highly controlled monkey studies present a unique resource that can be used to identify key pathways responsive to CR in primates, to uncover primate-specific aspects of the basic biology of ageing, and to determine molecular basis for nutritional modulation of health and ageing. Processes impacted by CR would be prime targets for the development of clinical interventions to offset age-related morbidity, and identification of factors involved in the mechanisms of CR will be pivotal in bringing these ideas to clinical research and human health care.

## Methods

### Animal care

Specifics of housing and animal care have been described in detail elsewhere^20^,^21^. Briefly, all animals are maintained according to the provisions of the Animal Welfare Act of 1966 (Public Law 89–544), plus its subsequent amendments, as well as the standards set forth in the document entitled ‘The Guide for the Care and Use of Laboratory Animals' (NIH Publication No. 85–23). Animals rooms are maintained at∼21 °C and 50–65% humidity. Room lighting is automatically controlled on a 12-h light, 12-h dark schedule. Animals are monitored daily for general health by dedicated animal care and research staff and routinely by veterinary staff. Studies at UW and NIA were conducted in accordance with protocols approved respectively by the University of Wisconsin–Madison Graduate School and National Institute on Aging Institutional Animal Care and Use Committees.

### Statistical analysis

The mortality analysis included all animals with known diagnoses or cause of death prior to July 2015. For time-to-event outcomes, the three most common methods were used: Kaplan-Meier product-limit method; Cox proportional hazard regression and parametric survival analysis assuming Weibull distributions^30^. Analyses were separated by site (UW; NIA), age-of-onset and sex. For both Kaplan-Meier and Cox proportional hazards (PH) regression, diet group (CR or control) was used as a predictor to test the effect of CR on mortality and morbidity (the onset of age-associated disease). Cox models were also used to estimate the HRs and 95% CIs. For the mortality analyses, age at death or current age for censored values was used as the time variable. Estimated median and mean lifespans were calculated using both Kaplan-Meier analysis and parametric survival analysis assuming a Weibull distribution. Bodyweight and food intake data were analysed using mixed linear models to account for dependency in the observations due to repeatedly measuring each animal over time. In the morbidity analyses, the age at which the animal experienced its first age-associated diagnosis was used as the time variable. Animals that had had not experienced an age-associated disease but died of an age-related cause were not censored and their age at death was used as the time variable. For the censored cases where the monkeys were alive and had not experienced an age-associated disease, current age was used as the time variable. For censored cases where monkeys had died but had not experienced an age-associated disease, age at death was used as the time variable. For all Cox models, the PH assumption was tested by first fitting a non-PH Cox regression with a CR by time interaction. The CR by time interactions were not significant in any model; thus, PH models were considered valid. All analyses were performed in SAS 9.3. The statistical significance level was set at *α*=0.05 for all analyses with no adjustments^54^. Multilevel modelling was conducted to explore relationships among parameters.

### Data availability

The data sets analysed during the current study are available from the corresponding authors on reasonable request.

## Additional information

**How to cite this article:** Mattison, J. A. *et al*. Caloric restriction improves health and survival of rhesus monkeys. *Nat. Commun.* **8,** 14063 doi: 10.1038/ncomms14063 (2017).

**Publisher's note**: Springer Nature remains neutral with regard to jurisdictional claims in published maps and institutional affiliations.

## Supplementary Material

Supplementary Information Supplementary figures and supplementary tables.

## Figures and Tables

**Table 1:** Study design.

**Site**	**Group**		**Control N**	**CR N**	**Age of onset**	**Genetic origin**
NIA	Male	Juvenile	10	10	1–2 years	Indian/Chinese
		Adolescent	12	10	3–5 years	Indian/Chinese
		Old	10	10	16–23 years	Indian/Chinese
	Female	Juvenile	9	9	1–3 years	Indian
		Adult	15	11	6–14 years	Indian/Chinese
		Old	8	7	16–21 years	Indian
						
UW	Male		23	23	7–14 years	Indian
	Female		15	15	9–15 years	Indian

**Table 2:** Diet composition at each location.

**Diet component**	**NIA**		**UW**		
	**Control/CR (% by weight)**	**Nutrient sources**	**Control (% by weight)**	**CR (% by weight)**	**Nutrient sources**
Protein	17.3	soybean and fish meal	13.13	13.13	lactalbumin
Carbohydrate	56.9	wheat, corn, sucrose (6.8%)	60.92	58.31	corn, sucrose (45%), dextrin
Fat	5.0	soy, corn, fish oils	10.6	10.6	corn oil
Fibre	6.5–9.0	cellulose	5.0	5.0	cellulose
Vitamins	∼140% RDA		100% RDA	∼130% RDA	
Calories kcal g^−1^	3.9		3.9	3.8	

**Table 3:** Survival estimates.

**Groups**			**KM**	**KM**	**KM**	**KM**	**Weibull**	**Weibull**
			**Median**	**IQR**	**Mean**	**SE**	**Median**	**IQR**
*All cause survival estimates*								
Males	UW	Control	26.11	6.82	25.28	1.03	25.75	5.57
		CR	28.32	6.19	26.86	1.23	27.63	8.04
	NIA J/A	Control	28.78	NE	26.00	1.50	29.22	14.31
		CR	26.31	13.06	23.71	1.91	25.86	17.05
	NIA old	Control	34.78	9.16	34.19	1.89	34.80	7.23
		CR	37.10	10.42	34.82	1.95	35.88	8.71
								
Females	UW	Control	23.86	7.92	23.56	1.29	24.57	8.62
		CR	29.68	15.60	25.78	2.18	27.49	14.37
	NIA J/A	Control	25.67	12.65	24.58	1.71	25.81	14.21
		CR	22.63	NE	19.79	1.20	23.53	15.25
	NIA old	Control	27.19	8.98	28.57	1.59	29.71	8.91
		CR	27.87	11.88	26.13	2.65	26.49	8.80

KM, Kaplan-Meier; IQR, interquartile range.
