# Supplementary material for: Caloric restriction improves health and survival of rhesus monkeys
Source: Nat Commun. 2017 Jan 17;8:14063. doi: 10.1038/ncomms14063 (PMC5247583; doi:10.1038/ncomms14063)
Supplement: Supplementary Information — Supplementary figures and supplementary tables. [file ncomms14063-s1.pdf]

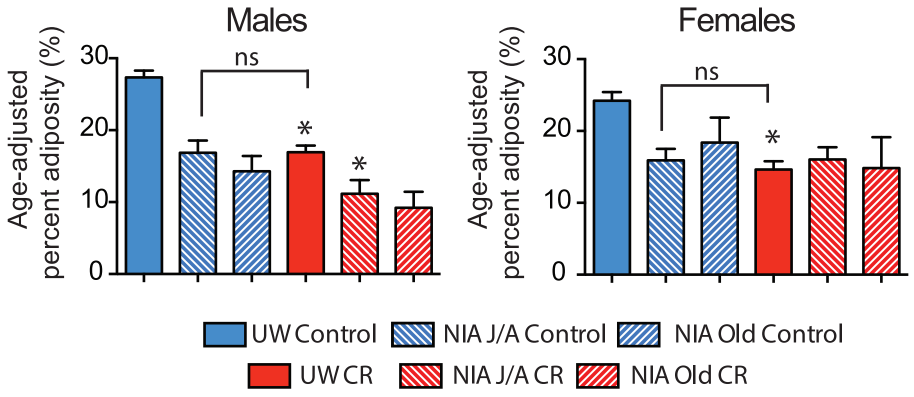

Supplementary Figure 1. Age-adjusted adiposity for males and females at UW and NIA. A main effect of age was on adiposity was detected for J/A and UW animals of both sexes but not for NIA Old. Data show adiposity as mean ± standard error of the mean statistically adjusted for age. A significant effect of diet is indicated by * ($p<0.05$: Type III test of fixed effects). A comparison of least squares means for J/A and UW aggregate data showed that adiposity of NIA J/A was not significantly different from UW CR for both sexes (per sex per group: UW n=23-39; J/A n=15-19; Old n=5-7).

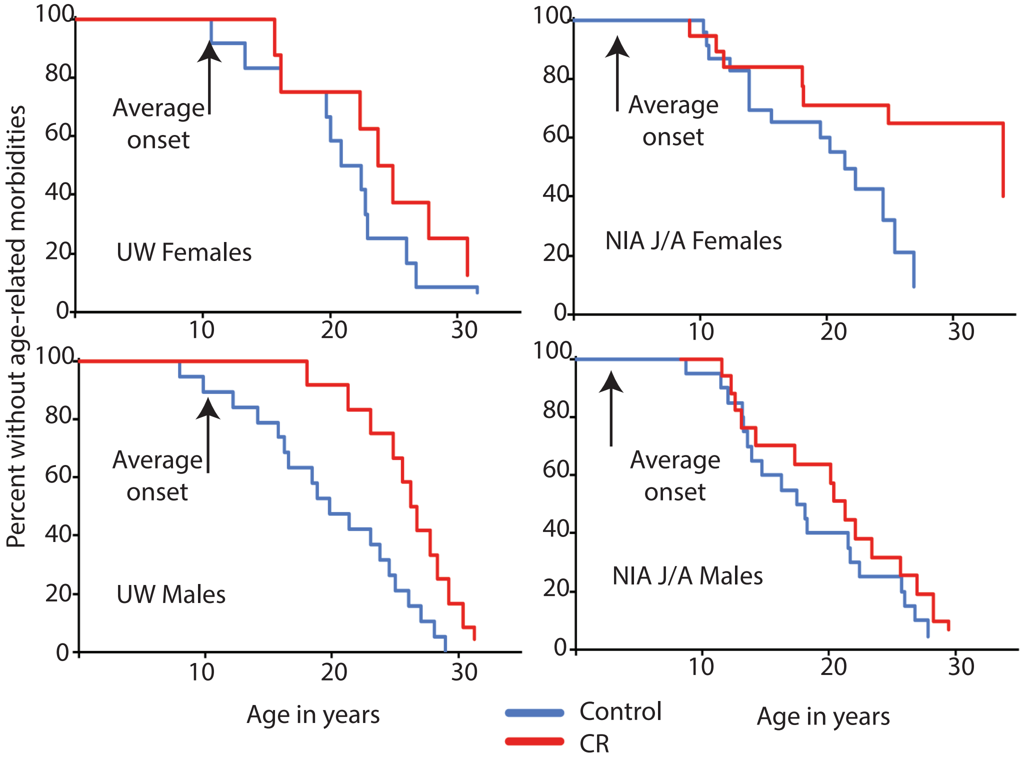

Supplementary Figure 2. Morbidity curves for females and males at UW and NIA. Data represent the first occurrence of any age-related disease, disorder, or condition for males and females separately. NIA animals are grouped by age where male J/A include juvenile and adolescent onset animals and female J/A include juvenile and adult onset animals. Statistics related to this figure are provided in Supplementary Table 4.

Supplementary Table 1: All-Cause Mortality

Group	KM p-value*	PH p-value#	HR	95 % CI
UW Combined	**0.0153**	**0.0168**	**1.865**	**1.119-3.108**
Male	0.0485	0.0524	1.939	0.993-3.786
Female	0.1740	0.1793	1.778	0.768-4.119
NIA J/A Combined	0.2784	0.2804	0.743	0.433-1.275
Male	0.3470	0.3506	0.692	0.319-1.499
Female	0.5098	0.5110	0.776	0.364-1.654
NIA Old Combined	0.9624	0.9624	1.017	0.511-2.022
Male	0.6868	0.6870	1.205	0.486-2.990
Female	0.4866	0.4890	0.688	0.239-1.985

*p-value from Kaplan-Meier product limit survival analysis. #p-value from Cox Proportional Hazard Regression Model. HR (Hazard Ratio) and Confidence Interval were obtained from the Cox PH Model.

Supplementary Table 2 Bodyweight data for control and CR monkeys from UW and NIA

Group (n)			Mean (Kg)	SD	Median (Kg)	IQR	p-value*
**11-13yrs**							
Males	UW	Control (17)	14.34	2.41	14.46	12.59-15.75	**0.0000**
		CR (18)	10.48	1.79	10.48	9.01-11.67	
	NIA J/A	Control (20)	11.15	3.17	9.96	9.07-12.52	**0.0041**
		CR (17)	8.52	1.71	8.15	7.44-9.51	
Females	UW	Control (10)	9.04	1.67	9.16	8.12-10.06	**0.0013**
		CR (7)	6.34	0.81	6.38	5.64-7.06	
	NIA J/A	Control (20)	6.23	1.71	5.72	4.92-7.54	0.3053
		CR (17)	5.70	1.30	5.28	4.59-7.02	
**18-20yrs**							
Males	UW	Control (21)	14.59	2.99	14.30	12.85-15.97	**0.0000**
		CR (20)	10.33	1.66	10.03	9.35-11.00	
	NIA J/A	Control (19)	12.68	3.79	12.21	10.21-13.97	**0.0305**
		CR (15)	10.16	2.30	9.74	8.19-11.62	
	NIA Old	Control (7)	10.31	1.19	10.02	9.50-11.00	0.2300
		CR (6)	9.36	1.51	9.30	8.45-9.80	
Females	UW	Control (12)	9.42	1.36	9.91	8.63-10.46	0.0066
		CR (11)	7.64	1.47	7.43	6.48-8.34	
	NIA J/A	Control (18)	7.66	2.24	7.81	5.50-9.58	0.3974
		CR (14)	7.02	1.86	7.13	5.12-8.66	
	NIA Old	Control (5)	6.94	1.11	6.83	6.55-8.00	0.2022
		CR (3)	5.64	1.48	4.97	4.60-6.30	
**25-27yrs**							
Males	UW	Control (12)	13.07	2.22	13.05	11.35-15.25	**0.0004**
		CR (13)	10.15	1.15	9.79	9.40-10.87	
	NIA J/A	Control (17)	12.64	4.06	11.77	9.06-14.86	**0.0230**
		CR (10)	9.19	2.47	8.24	7.10-10.99	
	NIA Old	Control (7)	10.83	2.06	11.18	8.95-12.47	0.2041
		CR (8)	9.21	2.55	9.29	7.27-10.62	
Females	UW	Control (7)	9.30	1.16	9.03	8.69-9.54	**0.0016**
		CR (10)	7.21	1.06	7.30	6.42-7.52	
	NIA J/A	Control (7)	7.41	1.45	7.76	6.25-8.27	0.3137
		CR (3)	6.40	1.10	6.56	5.13-7.34	
	NIA Old	Control (5)	6.86	0.64	6.86	6.31-7.49	0.9531
		CR (4)	6.89	1.09	6.80	6.32-7.49	

*p values calculated using mixed linear models

Supplementary Table 3: Statistics associated with Control and CR bodyweight comparisons by age category

**Group**			**P values***		
			11-13 years	18-20 years	25-27 years
Control	Females	UW vs J/A	**0.000**	**0.022**	**0.020**
		J/A vs Old	-	0.501	0.443
		UW vs Old	-	**0.003**	**0.002**
	Males	UW vs J/A	**0.002**	0.082	0.744
		J/A vs Old	-	0.121	0.277
		UW vs Old	-	**0.001**	**0.044**
CR	Females	UW vs J/A	0.241	0.374	0.271
		J/A vs Old	-	0.250	0.579
		UW vs Old	-	0.059	0.620
	Males	UW vs J/A	**0.002**	0.792	0.227
		J/A vs Old	-	0.446	0.989
		UW vs Old	-	**0.002**	0.257

*p values calculated using mixed linear models

Supplementary Table 4: Common pathologies in rhesus monkeys

Diseases & disorders	Specific conditions*
Cancer	Adenocarcinoma (intestinal, pancreatic, endometrioid), Hepatocellular carcinoma, Lymphoplasmacytic leukemia, Squamous cell carcinoma
Heart disease	Cardiomyopathy, Congestive heart failure, Fibrosis, Myocardial infarction
Metabolic disorders	Diabetic complications
Vascular complications	Stroke, Cerebral edema, Atherosclerosis
Inflammation/Immune dysfunction	Arthritis, Degenerative joint disease
Organ dysfunction	Liver failure, Kidney failure, Amyloidosis, Sarcopenia
Loss of resilience	Pneumonia, Septicemia, Abscess, Diverticulosis

*The conditions listed are among those identified on necropsy at both locations but not a comprehensive list of all pathologies identified.

Supplementary Table 5: Morbidity

Group	KM p-value*	PH p-value#	HR	95 % CI
UW Combined	**0.0003**	**0.0006**	**2.665**	**1.527-4.653**
Male	**0.0007**	**0.0013**	**3.401**	**1.614-7.168**
Female	0.1040	0.1113	2.091	0.844-5.181
NIA J/A Combined	**0.0107**	**0.0125**	**2.063**	**1.169-3.641**
Male	0.2631	0.2666	1.488	0.738-3.000
Female	**0.0127**	**0.0185**	**3.341**	**1.224-9.121**

*p-value from Kaplan-Meier product limit morbidity analysis. #p-value from Cox Proportional Hazard Regression Model. HR (Hazard Ratio) and Confidence Interval were obtained from the Cox PH Model.
